# Which One Would You Choose?—Investigation of Widely Used Housekeeping Genes and Proteins in the Spinal Cord of an Animal Model of Amyotrophic Lateral Sclerosis

**DOI:** 10.3390/neurosci6030069

**Published:** 2025-07-23

**Authors:** Aimo Samuel Christian Epplen, Sarah Stahlke, Carsten Theiss, Veronika Matschke

**Affiliations:** Department of Cytology, Institute of Anatomy, Medical Faculty, Ruhr-University Bochum, 44780 Bochum, Germany; aimo.epplen@rub.de (A.S.C.E.); sarah.stahlke@rub.de (S.S.); carsten.theiss@rub.de (C.T.)

**Keywords:** ALS, Wobbler mouse, neurodegenerative disease, calnexin, tubulin, actin

## Abstract

Amyotrophic lateral sclerosis (ALS) remains a progressive neurodegenerative disease, lacking effective causal therapies. The Wobbler mouse model harboring a spontaneous autosomal recessive mutation in the vacuolar protein sorting associated protein (Vps54), has emerged as a valuable model for investigating ALS pathophysiology and potential treatments. This model exhibits cellular and phenotypic parallels to human ALS, including protein aggregation, microglia and astrocyte activation, as well as characteristic disease progression at distinct stages. Exploring the underlying pathomechanisms and identifying therapeutic targets requires a comprehensive analysis of gene and protein expression. In this study, we examined the expression of three well-established housekeeping genes and proteins—calnexin, ß-actin, and ßIII-tubulin—in the cervical spinal cord of the Wobbler model. These candidates were selected based on their demonstrated stability across various systems like animal models or cell culture. Calnexin, an integral protein of the endoplasmic reticulum, ß-actin, a structural component of the cytoskeleton, and ß-tubulin III, a component of microtubules, were quantitatively assessed using quantitative reverse transcription-polymerase chain reaction (RT-PCR) for gene expression and Western blotting for protein expression. Our results revealed no significant differences in the expression of *CANX*, *ACTB*, and *TUBB3* between spinal cords of wild-type and Wobbler mice at the symptomatic stage (p40) at both the gene and protein levels. These findings suggest that the pathophysiological alterations induced by the Wobbler mutation do not significantly affect the expression of these crucial housekeeping genes and proteins at p40. Overall, this study provides a basis for further investigations using the Wobbler mouse model, while highlighting the potential use of calnexin, ß-actin, and ßIII-tubulin as reliable reference genes and proteins in future research to aid in the discovery for effective therapeutic interventions.

## 1. Introduction

Amyotrophic lateral sclerosis (ALS) is a neurodegenerative disease that currently lacks a definitive therapeutic approach for addressing its underlying causes. Patients face a progressive disease that begins with pain and fasciculations of the musculature and ends with extensive paralysis, including the respiratory muscles. At the end of the steadily progressive disease, death usually occurs after a few years [1]. The Wobbler mouse model is one of the tools used to study the pathophysiology and treatment options of the disease. The cause for the Wobbler disease is an autosomal recessive inherited gene defect in the vacuolar protein sorting-associated protein (Vps54), localized on chromosome 11, which occurred spontaneously in a C57BL/Fa mouse strain [2]. The *Vps54*-gene encodes part of the Golgi-associated retrograde protein complex (GARP), which is responsible for retrograde vesicle transport [2]. Homozygous Wobbler mice affected by the loss-of-function mutation exhibit impaired vesicle transport, endosomal accumulation and protein aggregation. Other pathomechanisms include mitochondrial dysfunction and impaired redox balance [3,4]. Ultimately, these impairments lead to death of upper and lower motor neurons [2,5,6]. The parallels between the Wobbler model and human ALS exist at both phenotypic and cellular levels. Cellularly, protein aggregation [7] and activation of microglia and astrocytes in the context of inflammatory processes are found in both species [8,9,10,11]. The similarity to human ALS in the mouse model concerns the characteristic course of the disease, which progresses from initial motor symptoms to paralysis [12]. In human ALS, the symptoms usually begin around the age of 50, progressing rapidly for three to five years until lethal outcome [1]. In Wobbler mouse model, this progression is markedly accelerated but follows a comparable pattern of symptom emergence and worsening. From birth, Wobbler animals develop similarly to control animals for three weeks. From p20, symptoms such as wobbly gait, head tremor, and muscle atrophy appear. In the stable clinical phase from p40 onwards, the symptoms increasingly intensify, and the degeneration of motor neurons increases [11,13]. This degenerative phase is characterized by multiple pathological processes in the cervical spinal cord, including the upregulation of apoptotic markers such as cleaved Caspase-3 and Bax, as well as components of the inflammasome pathway like NLRP3, indicating active neuroinflammation and cell death mechanisms [14,15]. Additionally, oxidative stress plays a significant role, evidenced by increased reactive oxygen species (ROS) levels due to Nmnat2 downregulation and defects in the glutathione system [4,16,17]. These findings underscore the complex molecular landscape of neurodegeneration in Wobbler mice at p40. Although the pathological state of the spinal cord at p40 is well documented on molecular and histological levels, systematic behavioral characterization of disease progression in Wobbler mice remains under-represented in the literature. While hallmark symptoms such as tremor, muscle atrophy, and gait instability are consistently reported, detailed quantitative assessments of motor performance (e.g., grip strength or coordination tests) are scarce. This gap further supports the need for robust molecular reference points when interpreting disease-related changes in gene and protein expression.

Working with the Wobbler mouse model to study pathomechanisms and discover new therapeutics involves the analysis of gene and protein expression, among many other methods. Quantitative reverse transcription-polymerase chain reaction (qPCR) and Western blotting are standard methods that usually require references at the mRNA or protein level to normalize and relate the values obtained. Several reference genes and proteins have been widely used in research due to their stable expression between individuals. In this study, we aim to test three established housekeeping genes and proteins on the cervical spinal cord of the Wobbler model to provide a basis for further investigations. We have selected calnexin, ß-actin, and ßIII-tubulin as possible candidates, which are already established in other systems, such as animal models or cell culture, and will now be tested in the spinal cord of the Wobbler model [18,19,20]. Calnexin (encoded by *CANX* gene) is an integral protein of the endoplasmic reticulum (ER) and acts as a chaperone. In the ER, membrane-bound calnexin binds to incompletely folded N-glycosylated proteins and assists their correct folding [21]. ß-Actin (encoded by *ACTB* gene) is a cytoskeleton structural protein found abundantly in all eukaryotic cells. It is highly conserved across species and is involved in essential biological processes such as cell migration, cell division, embryonic development, wound healing, and immune response. Its versatile functions contribute significantly to maintaining cell integrity, facilitating movement, orchestrating development, promoting tissue repair, and modulating immune reactions [22]. Tubulin, the monomeric protein component of microtubules, extends as long, stiff polymers through the cytoplasm, controlling the position of membrane-enclosed organelles and other cellular components. Tubulin is a dimer of two largely identical globular proteins called α-tubulin and β-tubulin. βIII-tubulin (encoded by *TUBB3* gene) is constitutively expressed in the central and peripheral nervous systems and in the testes, particularly in Sertoli cells. In addition, its expression can be induced in other tissues, both normal and neoplastic, exposed to a toxic microenvironment characterized by hypoxia and poor nutrient supply [23]. In a previous paper, it was investigated whether in the SOD1-ALS mouse model, among others, the housekeeping genes *ACTB* and *TUBB3* differ with regard to their expression in the different stages of the mouse model (early symptomatic, symptomatic, and terminal). It was found that at the mRNA level, the expression of *ACTB* changes during progression. At the protein level, ß-actin is stable during progression, whereas ß-tubulin changes its expression [24]. In this study, the expression levels of the candidates calnexin, ß-actin, and ßIII-tubulin will be compared between wild-type and Wobbler mice using qPCR and Western blot.

## 2. Materials and Methods

### 2.1. Animals

All animal procedures were conducted in strict compliance with the guidelines set by the German federal state of North Rhine-Westphalia and the European Communities Council Directive 2010/63/EU concerning the protection of animals used for scientific purposes. According to current German and European legislation, the removal of organs or cells from vertebrates for scientific purposes is not considered an animal experiment, if the animals have not been subject to surgical interventions or invasive treatments prior to sacrifice. Thus, the euthanization of mice intended for the removal of tissue does not need an approval or permission by local or governmental authorities. The mouse strain used was C57BL/Fa carrying the Wobbler mutation (homozygous) and wild types (homozygous) as control. Heterozygous mice were used for breeding. The mice were bred and genotyped as previously described [13]. All mice were kept at a 12 h day/night cycle in an open cage, in which food and water were available *ad libitum*. Cervical spinal cord tissue (C2–C7) from a total of 28 wild-type and 28 Wobbler mice each were analyzed; 12 mice were used for gene expression analysis and 16 for protein expression analysis. The mice carrying the mutation develop in a characteristic way: From birth, the animals develop like controls for three weeks. From p20, symptoms such as wobbly gait, head tremor, and muscle atrophy appear. In the stable clinical phase from p40 onwards, the propagation of symptoms stagnates [11,13]. Thus, p40 represents the time point in the disease course of mice that is most comparable to manifest ALS in humans.

### 2.2. Quantitative Reverse Transcription-Polymerase Chain Reaction

Total RNA (tRNA) was extracted from the cervical spinal cord tissue of healthy wild-type and Wobbler mice at p40 using the NucleoSpin miRNA Kit (Macherey-Nagel, Düren, Germany, Cat# 740971), following the original manufacturer’s protocol. The synthesis of cDNA was carried out using a Reverse Transcription System (Promega, Madison, WI, USA, Cat# A3500), as outlined in the manufacturer’s protocol, with 1 μg of tRNA and oligo(dT) primer. The resulting cDNA was stored at −20 °C until further use. Standard quantitative reverse transcription-polymerase chain reaction (qRT-PCR) was performed on a CFX96 Real-Time PCR Detection System (Bio-Rad, Hercules, CA, USA) using GoTag qPCR Master Mix (Promega, Walldorf, Germany, Cat# A6001). The primer sequences for *CANX* were sense: 5′-ATG ACT GGG ATG AAG ACG CC-3′ and antisense: 5′-GTC GTC TAG CCA GCC TTC AG-3′. The primer sequences for *ACTB* were sense: 5′-CAG CCT TCC TTC TTG GGT ATG -3′ and antisense: 5′-GGC ATA GAG GTC TTT ACG GAT -3′. The primer sequences for *TUBB3* were sense: 5′-GCC ATT CTG GTG GAC TTG GA-3′ and antisense: 5′-GTC GGG CCT GAA TAG GTG TC -3′. In order to use exactly the same amount of cDNA in each reaction for qPCR, the concentration of cDNA after reverse transcription was measured in triplicate with a spectrophotometer (NanoDrop One, Thermo Scientific, Waltham, MA, USA), and the mean value was calculated. The cDNA amount was adjusted to 30 ng/µL. The newly prepared concentration of all samples was determined again (triple measurement with subsequent averaging). To allow for a final dilution with a lower dilution factor to increase accuracy, all samples were afterwards diluted to a final concentration of 20 ng/µL. After this step, the concentration was determined again. Once the exact value of the cDNA concentration in each sample was determined, it was possible to ensure that exactly 80 ng of cDNA was used in each reaction in the subsequent qPCR. Maximum precision in sample preparation is necessary to enable comparability. On each 96-well qPCR plate, samples of 4 wild-type and 4 Wobbler mice were measured in triplicate. In total, three 96-well-qPCR plates were used, analyzing 12 individual mice of each genotype. Thus, in total, 36 Cycle Threshold (C_t_) values were collected per genotype.

### 2.3. Semiquantitative Protein Expression Analysis via Western Blot

Protein isolation from cervical spinal cord tissue was performed using RIPA buffer (150 mM NaCl, 20 mM Tris-HCl, 0.1% sodium dodecyl sulfate, 1% Triton-X100, 1% sodium deoxycholate, 1 mM Na_2_EDTA). The tissue was stored on ice and supplemented with 10 µL RIPA buffer per 1 mg and 1% protease inhibitor cOmplete EDTA-free (Roche Diagnostics, Mannheim, Germany, Cat# 05056489001). It was homogenized using a micropestle to release the protein. Finally, the samples were centrifuged for 10 min at 4 °C and 11,000× *g* to sediment unlysed cells and cell debris. The supernatant was transferred to a new reaction tube. The concentration of the extracted proteins was determined using the Pierce^TM^ BCA Protein Assay Kit (Thermo Fisher Scientific, Waltham, MA, USA, Cat# 23225). For subsequent analysis, 20 μg of total protein was loaded per lane. In addition, 10% Mini-PROTEAN^®^ TGX Stain-Free™ Protein Gels (Bio-Rad, Hercules, CA, USA, Cat#4568034) were used for the separation of proteins. The blocking step was conducted at room temperature in 1× phosphate-buffered saline (PBS) supplemented with 1% RotiBlock (Roth, Karlsruhe, Germany, Cat# A151) for a minimum duration of 1 h. The subsequent procedure for protein expression analysis without further reference proteins was performed using stain-free imaging technology and total protein normalization. The Precision Plus Protein™ Unstained Protein Standard and Precision Plus Protein Dual Color Standard (Bio-Rad, Hercules, CA, USA, Cat#1610363, and Cat#1610374) were used. The following antibodies were used for protein detection: primary rabbit polyclonal IgG anti-ß-actin (20–33) antibody (1:250; Sigma-Aldrich, Schnelldorf, Germany, RRID: AB_476738, Cat# A5060), and secondary horseradish peroxidase-conjugated goat anti-rabbit IgG antibody (1:5000; Santa Cruz Biotechnology, Dallas, TX, USA, RRID: AB_631748, Cat# sc-2054) for detecting ß-actin. Primary mouse polyclonal IgG anti-ß-tubulin antibody (1:200; Sigma-Aldrich, Schnelldorf, Germany, RRID: AB_477577, Cat# T4026) and secondary horseradish peroxidase-conjugated donkey anti-mouse IgG antibody (1:5000; Santa Cruz Biotechnology, Dallas, Texas, USA, RRID: AB_641170, Cat# sc-2314) were used to detect ßIII-tubulin. Further, a primary antibody anti-calnexin polyclonal IgG antibody (Novus Biologicals, Wiesbaden, Germany, RRID: AB_10002123, Cat# NB100-1965) derived from rabbit and secondary horseradish peroxidase-conjugated goat anti-rabbit IgG antibody (1:5000; Santa Cruz Biotechnology, Dallas, TX, USA, RRID: AB_631748, Cat# sc-2054) were employed in the experiment. To visualize the protein bands, Western blotting Luminol Reagent (Santa Cruz Biotechnology, Dallas, Texas, USA, Cat# sc-2048) was used. The individual samples were normalized to the total applied protein amount in the respective lane. To use two different primary antibodies on one blot paper, the two target proteins had to be sufficiently separated in size. The blots were cut with a safe distance between the proteins to be analyzed and treated separately in subsequent steps. In each blot, each individual lane was normalized to a selected lane of the same blot. The software analysis with Image Lab 6.1 software (Bio-Rad, Hercules, CA, USA) was performed strictly according to the manufacturer’s instructions.

### 2.4. Statistical Analysis

Data were statistically analyzed with GraphPad Prism 6.1 software (GraphPad Software, San Diego, CA, USA). Kolmogorov–Smirnov normality test was used to confirm normal distribution. Data were tested for significance using Student’s t test. Results with *p* < 0.05 were considered statistically significant.

## 3. Results

### 3.1. Similar Gene Expression in the Cervical Spinal Cord of Wild Type and Wobbler Mice at the Stable Clinical Phase

**Figure 1:**
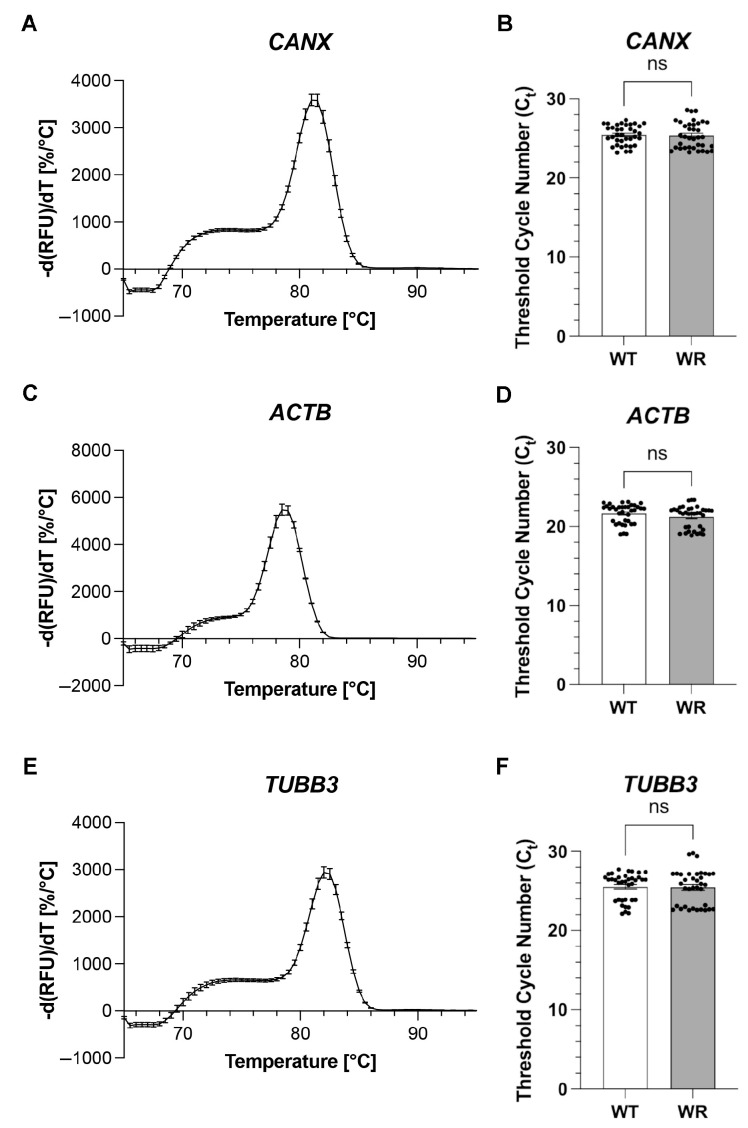
**Analysis of gene expression in the cervical spinal cord of wild-type (WT) and Wobbler (WR) mice.** Representative melting curves for qPCR products of (**A**) CANX, (**C**) ACTB, and (**E**) *TUBB3* mRNA, confirming the specificity of primer pairs by generating a single peak for each gene. Quantification of gene expression, represented as cycle threshold (Ct) values, for (**B**) CANX, (**D**) ACTB, and (**F**) TUBB3, in WT and WR mice at the stable clinical phase (p40). The data indicate similar gene expression levels between WT and WR mice for all three reference genes, with no significant differences observed. Bars represent mean ± SEM, with all individual data points displayed. Data were tested for significance using Student‘s *t*-test. The number of animals per genotype and tested gene is n = 12.

In this study, we investigated the expression of three well-established reference genes—calnexin, ß-actin, and ßIII-tubulin—in the cervical spinal cord of wild-type and Wobbler mice at the stable clinical phase (p40). We quantitatively assessed the gene expression of *CANX*, an integral protein of the endoplasmic reticulum, *ACTB*, a structural component of the cytoskeleton, and *TUBB3*, a microtubule component, using quantitative reverse transcription-polymerase chain reaction (RT-PCR). To verify the specificity of our primers in generating a single targeted product, we analyzed the melting curves of the qPCR products. This analysis revealed that each primer pair produced a single peak in the melting curve (Figure 1A,C,E). The quantification of gene expression revealed Ct values, with a mean of 25.2 for the WT and 25.0 for the WR regarding *CANX*. For *ACTB*, the mean CT values were 21.0 for WT and 20.7 for WR. For *TUBB3*, the corresponding values were 25.1 and 24.9 for WT and WR, respectively. Classical *t*-tests analyzing gene expression levels of *CANX*, *ACTB*, and *TUBB3* indicated no significant differences between wild-type and Wobbler mice.

**Figure 2:**
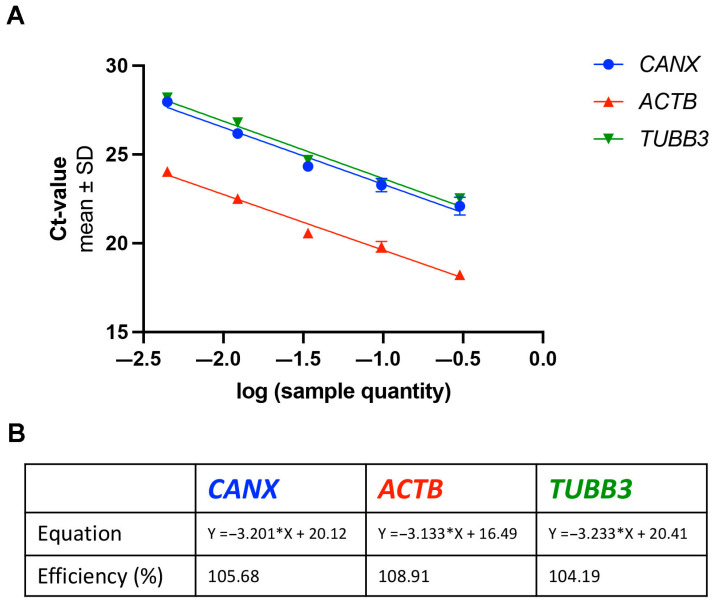
**Standard curves and primer efficiency for the housekeeping genes *CANX*, *ACTB*, and *TUBB3*.** The diagram in (**A**) shows the standard curves for the investigated housekeeping genes. The logarithm of the sample concentration is plotted on the x-axis, while the mean values of the Ct values ± SD are plotted on the y-axis. The standard curves are shown in the following colors: *CANX* is shown in blue, *ACTB* in red, and *TUBB3* in green. Using linear regression analysis, we derived the equations representing these curves, which were then used to determine the primer efficiencies. The equations for the respective standard curves and the calculated efficiency are shown in (**B**).

Figure 2 illustrates the standard curves for the analyzed housekeeping genes. By linear regression analysis, equations representing these curves were developed and utilized to determine the primer efficiencies. The derived equations are as follows: Y = −3.201X + 20.12 for *CANX*; Y = −3.133X + 16.49 for *ACTB*; and Y = −3.233X + 20.41 for *TUBB3*. These equations enabled the calculation of the primer efficiencies, which were found to be 105.68% for *CANX*, 108.91% for *ACTB*, and 104.19% for *TUBB3*. The inclusion of these standardized curves and the calculated primer efficiencies will improve the reliability of our gene expression analysis.

### 3.2. Consistent Protein Expression in the Cervical Spinal Cord of Wild-Type and Wobbler Mouse Models

**Figure 3:**
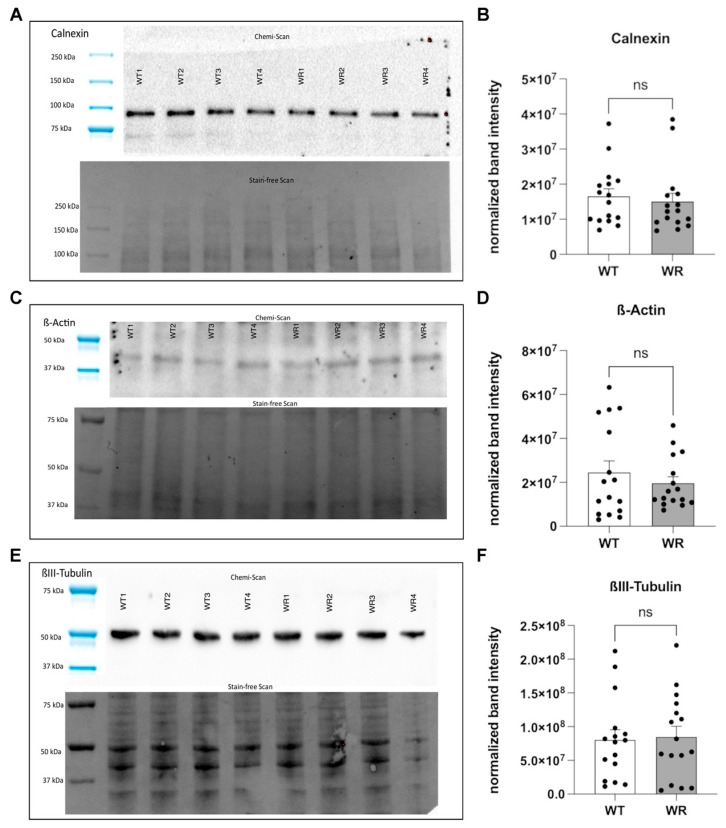
**Protein expression analysis of candidate reference proteins in wild-type (WT) and Wobbler (WR) mice**. Representative blots and images of the stain free gels used für semiquantitative protein expression analysis of (**A**) calnexin (~95 kDa), (**C**) ß-actin (~42 kDa), and (**E**) ßIII-tubulin (~50 kDa). Semiquantitative analysis of protein expression levels in the cervical spinal cord of p40 WT and WR mice for (**B**) Calnexin, (**D**) ß-Actin (~42 kDa), and (**F**) ßIII-Tubulin. Stain-free scan images were used for normalization of the respective protein of interest. Bars represent mean ± SEM, with all individual data points displayed. Data were tested for significance using Student‘s *t*-test. The number of animals per genotype and per tested protein is n = 16.

Western blot analyses were performed to evaluate whether protein expression levels are consistent across various homozygous genotypes at p40, thereby facilitating their use as references in semi-quantitative assessments of protein expression (Figure 3). This method involved assessing the positioning of protein bands relative to molecular weight markers on the Chemi scan. Notably, the expected molecular weight of calnexin is approximately 95 kDa, typically appearing just below the 100 kDa marker band (Figure 3A). Additionally, bands for ß-actin (Figure 3C) and ßIII-tubulin (Figure 3E), with molecular weights around 42 kDa and 50 kDa, respectively, were expected to align closely with their corresponding markers.

A distinct feature of stain-free scans is their ability to visualize total protein content on a single blot. This enables the normalization of detected proteins against the total protein transferred, thus allowing quantification without dependency on control protein expression. In this context, proteins are visualized not as discrete bands but as vertical “columns” of separated proteins on the scan (Figure 3A,C,E lower panel). These columns are essential for normalizing the expression levels of specific proteins such as calnexin, ß-actin, and ßIII-tubulin. Despite the observed variability in the normalized volume of bands, which suggests a potential divergence from protein expression patterns, statistical analysis using Student’s *t*-tests indicated no significant differences in protein expression between wild-type and Wobbler mice (Figure 3B,D,F). This outcome highlights the effectiveness of stain-free scanning technology in providing reliable, semi-quantitative data on protein expression across different genetic backgrounds, establishing its utility in molecular biology research.

## 4. Discussion

The study aimed to assess the suitability of calnexin, ß-actin, and ßIII-tubulin as reference genes and proteins in the Wobbler mouse model of neurodegeneration. This study serves to both support previous studies on Wobbler mice in which certain housekeeping genes and proteins were used, and to support and provide a firm basis for future studies. The results demonstrated that the expression levels of these genes and proteins remained stable between the wild-type and the Wobbler mice at the investigated time point p40. This finding provides support for utilizing these candidates as reliable reference markers in expression studies related to ALS and other neurodegenerative disorders.

Given that ALS in humans is an age-associated disease, the use of an early postnatal time point such as p40 in mice requires careful justification. However, the Wobbler mouse model represents a spontaneous, non-transgenic form of motor neuron degeneration that is characterized by early onset and rapid disease progression [24,25]. Clinical symptoms such as tremor, gait instability, and muscle atrophy consistently appear around postnatal week 3 (approx. p20), with a fully symptomatic disease phase typically reached between postnatal week 6–8 (approx. p40–p60), as confirmed by several independent studies [24,25,26,27,28]. Although the Wobbler model does not replicate the age-related onset of ALS in humans, it provides a well-defined window into early disease mechanisms. We therefore selected p40 as the optimal time point to assess reference gene and protein stability, as it represents a phase in which neurodegeneration is fully manifest. Although behavioral scoring remains underrepresented in the literature, the pathological state of the spinal cord at this stage has been consistently documented by histological and molecular markers, underscoring the relevance of p40 for molecular normalization studies in the Wobbler model. Our findings thus help to establish a reliable molecular baseline at this clinically relevant stage, particularly in the context of future studies that aim to correlate behavioral phenotypes with gene and protein expression.

Reference genes and housekeeping proteins play a crucial role in normalizing expression data and ensuring accurate comparisons between different samples [29,30]. In this study, the selection of appropriate references is particularly important because the Wobbler mutation affects vesicle trafficking, endosomal accumulation, and protein aggregation, which could potentially influence the expression of many genes and, therefore, also affect protein levels. By validating the stability of these reference genes and proteins, this study provides a valuable resource for future investigations using the Wobbler mouse model. It is likely that the functions of calnexin, ß-actin, and ßIII-tubulin are of such central importance to basic cellular homeostasis that their expression remains unaffected even in the presence of the pronounced neurodegenerative phenotype caused by the Wobbler mutation. Previous work by Usarek and Kozakiewicz (2017) has already examined the stability of *ACTB* in human lymphocytes under various experimental conditions. While their study provides valuable insight into *ACTB* regulation in peripheral human cells [31], our study expands on this knowledge by confirming *ACTB* (alongside *CANX* and *TUBB3*) as stable references in a disease-relevant, central nervous system tissue in vivo. Given the distinct species, tissue type, and disease background, such comparative validation is essential for ensuring reliable data normalization in neurodegenerative models like the Wobbler mouse.

The first transgenic mouse model of ALS was established on the basis of a mutation in the SOD1 gene: in position 93, glycine is substituted by alanine [32]. Mutations in this gene are present in some cases of familial ALS. In about 2–3% of sporadic ALS (sALS), mutations in the SOD1 gene are also found [33]. The mSOD1-G93A model has been used, among various other approaches, to describe the possible pathogenesis of cellular dysfunction as well as the non-cell autonomous nature of ALS [34]. There are now over ten models involving different mutations in the SOD1 gene [35]. The SOD1 mouse model depicts rapid degeneration of motor neurons leading to paralysis and death within the first five months of life [34,36]. Studies that have examined housekeeping genes and possible reference proteins in the SOD1-G93A mouse model can now be compared with the current study on the Wobbler model. While our study investigates different transcriptional and translational references compared to wild types, a previous study in the SOD1-G93A model focused more on reference genes and proteins that are stable between different stages of the disease to investigate disease progression [12]. Differences in the results can indeed be observed. The housekeeping genes and proteins constancy in the SOD1 mouse was very limited. At the RNA level, alterations in *ACTB* expression were observed throughout the progression. However, at the protein level, ß-actin exhibited stability over the course of progression, whereas ßIII-tubulin displayed variations in its expression. However, since our department usually focuses on the wild type vs. Wobbler comparison at time point p40, we chose this approach for the study design. Should the investigation of pathomechanisms in the course of the disease be the aim of the study, analyzing reference genes and proteins at different time points could be considered in future studies. There is condensed evidence that actin plays a significant role in aging [37]. These changes in ß-actin function and expression may explain the results in the SOD1 mouse, whereas ß-actin may well be used in the Wobbler mice at the age of p40. To set the preceding reference to the SOD1 mouse model in relation to the Wobbler mouse model, brief comparative information between these two models follows. Both models, Wobbler and SOD1, show similarities to human ALS at the cellular and phenotypic level, such as protein aggregation and inflammatory processes. In the Wobbler model, the disease is caused by a gene defect in the Vps54 gene, which leads to vesicle transport defects and ultimately results in motor neuron death [2,5,6]. In the SOD1-G93A mouse model, a mutation in the SOD1 gene is responsible for protein misfolding and aggregation, which also leads to motor neuron degeneration [32,38].

Both models show similar clinical courses, beginning with motor symptoms and progressive paralysis. However, there are also differences between the models. For example, disease progression in the Wobblers takes 40 days after birth (p40) to become completely symptomatic. In the SOD1 mice, however, the terminal phase occurs after 4 months [14]. The Wobbler mice also show defects in spermatogenesis that are not observed in human ALS [12,39,40]. The SOD1 model exhibits unstable copy numbers in the genome, which can lead to variable disease expression [41,42]. The convergence and divergences of findings between the Wobbler mouse model and the SOD1 model emphasizes the necessity of considering multiple animal models to comprehensively understand the complexity of ALS pathology and identify potential therapeutic targets.

## 5. Conclusions

Our study on the Wobbler mouse model has demonstrated the stability and reliability of calnexin, ß-actin, and ßIII-tubulin as reference genes and proteins for gene and protein expression studies in ALS research. Despite the significant pathophysiological alterations induced by the Wobbler mutation, the expression levels of these markers remained consistent between wild-type and Wobbler mice at a key stage of the disease (p40). This finding underscores the potential of these markers to serve as robust references in the quantitative analysis of gene and protein expression, facilitating accurate comparisons across studies focused on ALS. By validating these housekeeping genes and proteins in the cervical spinal cord of Wobbler mice as consistent references, we enable more detailed research into the molecular foundations of ALS using the Wobbler mouse. In moving forward, it will be essential to continue exploring the dynamics of these and other potential reference markers at various stages of disease progression and in response to therapeutic interventions. Thus, this study lays a solid groundwork for future explorations aimed at unraveling the complex molecular landscape of ALS, offering hope for the development of effective treatments for this devastating condition.

## Data Availability

All data supporting the findings of this study are included within the manuscript.

## Abbreviations

The following abbreviations are used in this manuscript:

ALS	Amyotrophic lateral sclerosis
Vps54	Vacuolar protein sorting-associated protein 54
GARP	Golgi-associated retrograde protein complex
ER	Endoplasmic reticulum
CANX	Calnexin gene
ACTB	Actin beta gene
TUBB3	Tubulin beta 3 class III gene
qPCR	Quantitative polymerase chain reaction
mRNA	Messenger ribonucleic acid
tRNA	Total RNA
qRT-PCR	Quantitative reverse transcription polymerase chain reaction
PBS	Phosphate-buffered saline
SDS	Sodium dodecyl sulfate
EDTA	Ethylenediaminetetraacetic acid
WT	Wild type
WR	Wobbler
Ct	Cycle threshold
SD	Standard deviation
SEM	Standard error of the mean
SOD1	Superoxide dismutase 1
sALS	Sporadic amyotrophic lateral sclerosis
BCA	Bicinchoninic acid
RRID	Research resource identifier
